# Comparative analysis of chloroplast genomes of kenaf cytoplasmic male sterile line and its maintainer line

**DOI:** 10.1038/s41598-021-84567-1

**Published:** 2021-03-05

**Authors:** Danfeng Tang, Fan Wei, Ruiyang Zhou

**Affiliations:** 1 College of Agriculture, Guangxi University, Nanning, 530004 China; 2 Guangxi Key Laboratory of Medicinal Resources Protection and Genetic Improvement, Guangxi Botanical Garden of Medicinal Plants, Nanning, 530023 China

**Keywords:** Biotechnology, Genetics, Molecular biology, Plant sciences

## Abstract

Kenaf is a great source of bast fiber and possesses significantly industrial interests. Cytoplasmic male sterility (CMS) is the basis of heterosis utilization in kenaf. Chloroplast, an important organelle for photosynthesis, could be associated with CMS. To understand the phylogenetic position and molecular basis of kenaf CMS from the perspective of chloroplast, the chloroplast (cp) genomes of the CMS line P3A and its maintainer line P3B were characterized and their comparative analysis was also performed. In this study, the chloroplast genomes of P3B and P3A were sequenced with 163,597 bp and 163,360 bp in length, respectively. A total of 131 genes including 85 protein coding genes (PCGs), 38 transfer RNA (tRNA) genes, and 8 ribosome RNA (rRNA) genes were annotated in P3B, while 132 genes containing 83 PCGs, 41 tRNA genes, and 8 rRNA genes were found in P3A. The phylogenetic tree revealed that kenaf was closely related to *Hibiscus syriacus* and *Abelmoschus esculentus*. Further analysis of single nucleotide polymorphism (SNP) and insertion and deletion (InDel) showed that compared with P3B, a total of 22 SNPs and 53 InDels were detected in gene coding region, gene intron, and intergenic regions of P3A. Remarkably, a total of 9 SNPs including 6 synonymous SNPs and 3 nonsynonymous SNPs were found in *psbK*, *atpA*, *rpoC2*, *atpB*, *rpl20*, *clpP*, *rpoA*, and *ycf1*. The present study provided basic information for further study of kenaf CMS mechsnism.

*Hibiscus genus* plants belong to the Malvaceae family of angiosperms to which other genera such as *Sterculia*, *Dombeya,* and *Pavonia* also belong. Kenaf (*Hibiscus cannabinus* L., 2n = 2x = 36) is a member of the *Hibiscus genus* with potential industrial and commercial interests and identified to be an excellent source of cellulosic fiber originated from bast or stalk for paper industries^1,2^. In addition, uses of kenaf fiber are not only limited to textile, but also equally important for new materials industries, such as building materials, adsorbents, and composites using new and recycled plastics, etc.^3^. In the recent past, kenaf seeds have been proved to be potential uses in chemical and bio-energy industries^4,5^.

Chloroplasts are present in photosynthetically active green tissues^6,7^ and display a conserved structure of a circular double-stranded DNA molecule^8^. Cytoplasmic male sterility (CMS) is an important agronomic character, which is widely utilized for F1 hybrid breeding^9^. Since the discovery of kenaf CMS, achievements have been made on the mechanism of CMS in kenaf. Up to now, several studies are performed on kenaf CMS mechanism^10^. However, the exact mechanism of kenaf CMS has not been fully elucidated. It is generally believed that cytoplasmic male sterility is closely related to mitochondria. Nevertheless, studies also demonstrated that the chloroplasts might be associated with plant CMS^6,11,12^. Therefore, in the analyses of the molecular mechanism of kenaf CMS, we should pay attention to the chloroplast genome. However, at present, little is known about the chloroplast genome information of kenaf CMS line and its maintainer line as well as the relationship between the chloroplast genome and kenaf CMS.

Here, we reported the complete cp genome sequences of the kenaf CMS line P3A and its maintainer line P3B by employing the Illumina Hiseq and PacBio platforms. The comparative analysis of the chloroplast genomes among Malvales was performed. SNP and Indel between the two lines were also detected, analyzed, and validated. This study characterized the chloroplast genomes of P3A and P3B and unveiled their discrepancies, which provided basic data for further study of kenaf CMS mechanism.

## Results

### Genome sequencing and assembly

The DNA bands of P3A and P3B were clear and the DNA was free of protein, pigment, and other impurities (Supplementary Fig. S1). In addition, OD260/280 and OD260/230 were about 1.8 and 2.0, respectively. It was indicated that the DNA quality, concentration, and total amount of DNA met the requirements of subsequent experiments (Supplementary Table S1). Then the chloroplast genomes of kenaf CMS line P3A and its maintainer line P3B were sequenced using Illumina Hiseq and PacBio platforms. About 6.3G and 3.7G raw data were generated while 6.1G and 3.5G clean data were produced in P3B and P3A, respectively. Q30 ratio reached 95.31% and 94.12% in P3B and P3A, respectively, indicating that the data was reliable (Supplementary Table S2).

**Figure 1:**
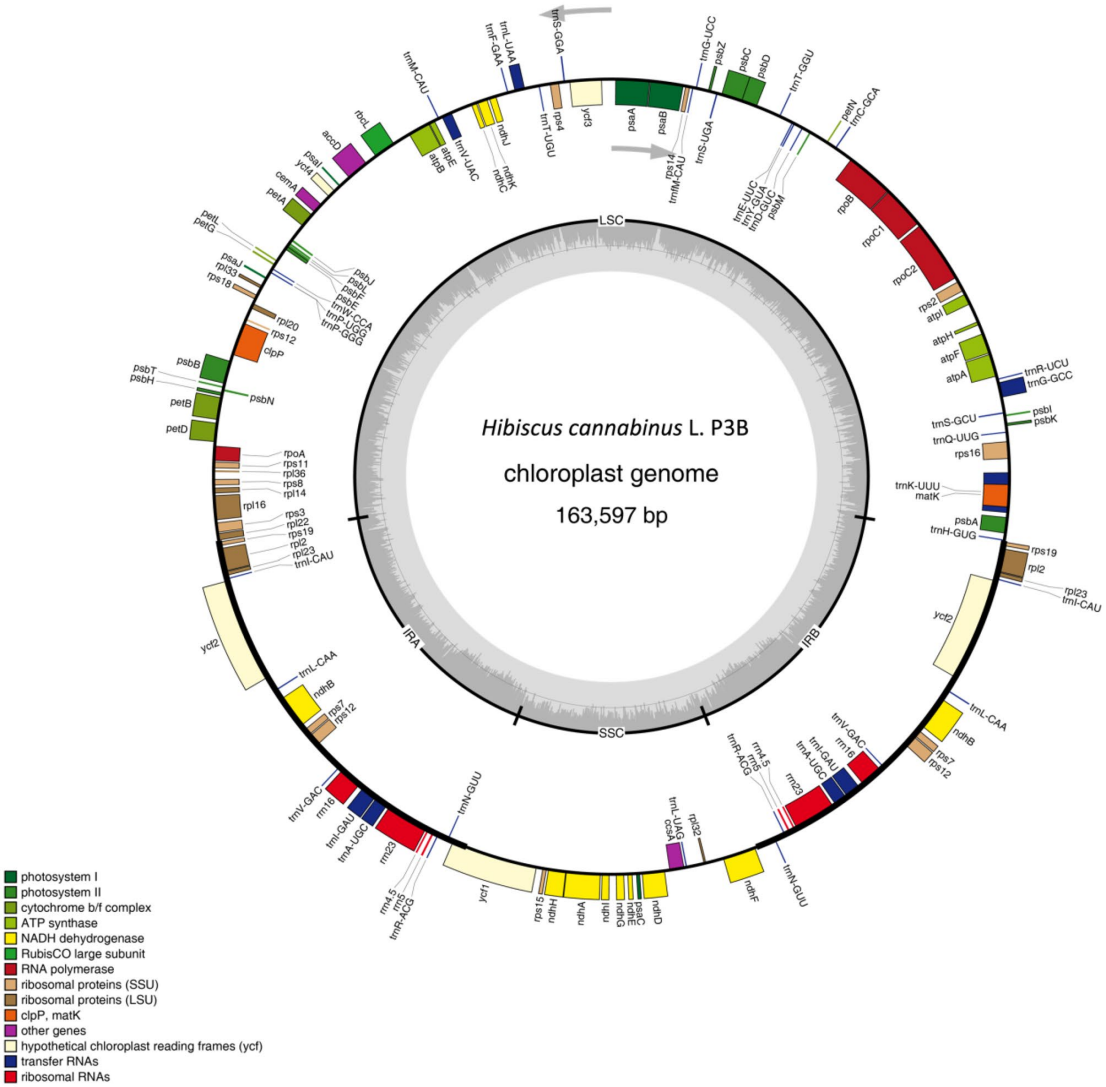
Circular gene map of *Hibiscus cannabinus* maintainer line (P3B). Genes drawn within the circle are transcribed clockwise, while those drawn outside are transcribed counter clockwise. Genes are colour-coded according to their functional groups.

**Figure 2:**
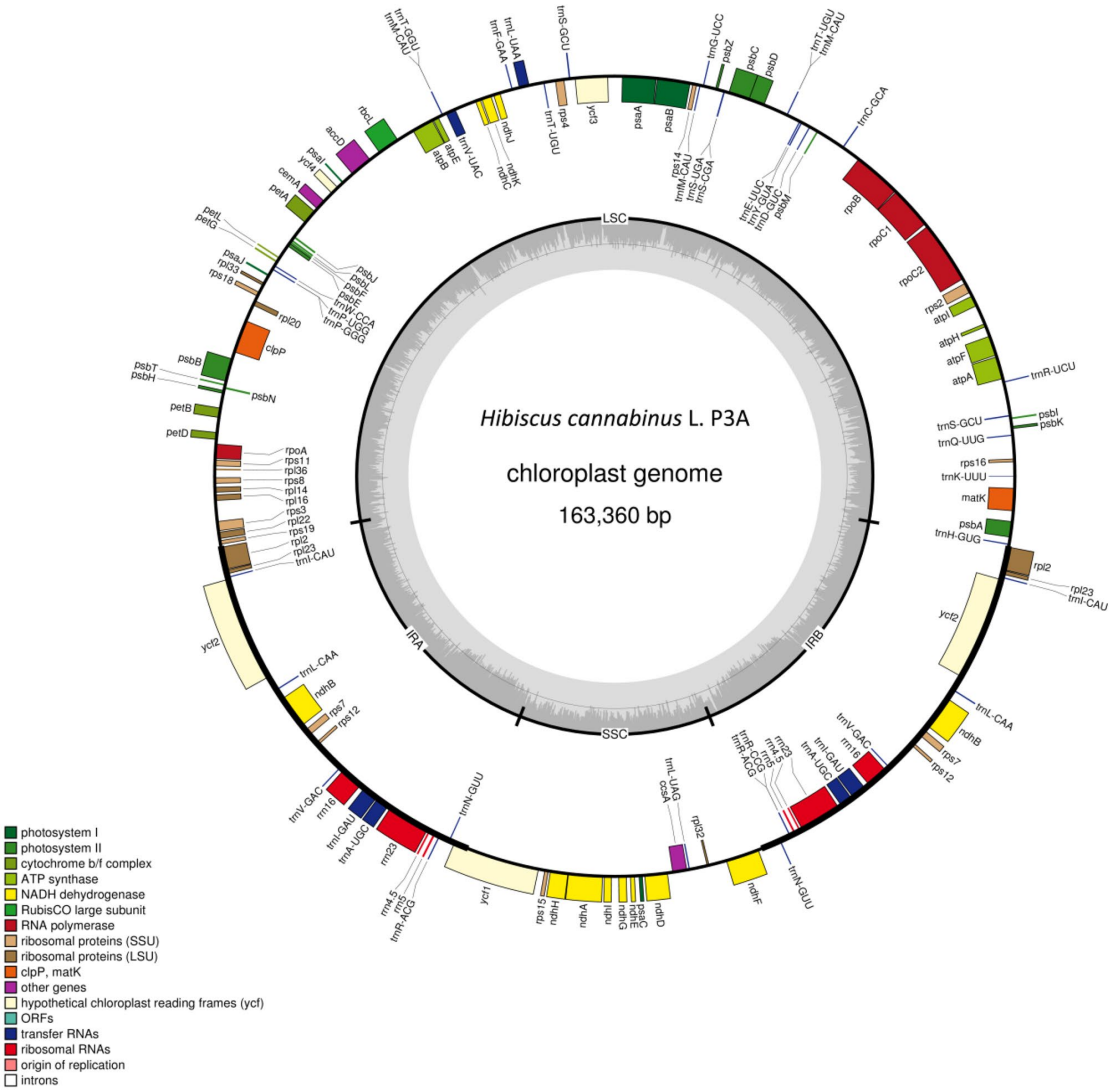
Circular Gene map of *Hibiscus cannabinus* CMS line (P3A).

The chloroplast genomes of P3B and P3A were assembled into circular molecule with a total length of 163,597 bp (Fig. 1) and 163,360 bp (Fig. 2), respectively. The chloroplast genome of P3B was 237 bp longer than that of P3A. The assembled genome sequences were deposited in GenBank with accession number MW446503 (P3B) and MW446504 (P3A).

### General features of kenaf P3B and P3A chloroplast genomes

In this study, the gene number, gene total length, gene average length, gene length/genome, and GC content were noted as 85, 79151 bp, 931 bp, 48.38%, and 36.55% in P3B, respectively. However, in P3A all those were recorded as 83, 87032 bp, 1049 bp, 53.28, and 36.57%, respectively (Table 1; Supplementary Table S3). The chloroplast genomes of P3B and P3A were observed to contain 38 and 41 tRNA genes, respectively. Each chloroplast genome had 8 rRNA genes (Supplementary Table S4). Two protein coding genes, *rps19-D2* and *petN*, were absent and three additional tRNA genes were observed in P3A.

**Table 1:** List of protein-coding genes present in *Hibiscus cannabinus.* L (P3B) chloroplast genome. “*”represented genes were absent in cp genome of P3A.

Category	Gene group	Gene name					
Genes for photosynthesis	Subunits of photosystem I	*psaA*	*psaB*	*psaC*	*psaI*	*psaJ*	*ycf3*
		*ycf4*					
	Subunits of photosystem II	*psbA*	*psbB*	*psbC*	*psbD*	*psbE*	*psbF*
		*psbH*	*psbI*	*psbJ*	*psbK*	*psbL*	*psbM*
		*psbN*	*psbT*	*psbZ*			
	Subunits of ATP synthase	*atpA*	*atpB*	*atpE*	*atpF*	*atpH*	*atpI*
	Subunits of cytochrome	*petA*	*petB*	*petD*	*petG*	*petL*	petN*
	Large subunit of Rubisco	*rbcL*					
	Subunits of NADH dehydrogenase	*ndhA*	*ndhB*	*ndhB-D2*	*ndhC*	*ndhD*	*ndhE*
		*ndhF*	*ndhG*	*ndhH*	*ndhI*	*ndhJ*	*ndhK*
Self-replication	Small subunit of ribosome	*rps2*	*rps3*	*rps4*	*rps7*	*rps7-D2*	*rps8*
		*rps11*	*rps12*	*rps12-D2*	*rps14*	*rps15*	*rps16*
		*rps18*	*rps19*	rps19-D2*			
	Large subunit of ribosome	*rpl2*	*rpl2-D2*	*rpl14*	*rpl16*	*rpl20*	*rpl22*
		*rpl23*	*rpl23-D2*	*rpl32*	*rpl33*	*rpl36*	
	DNA-dependent RNA polymerase	*rpoA*	*rpoB*	*rpoC1*	*rpoC2*		
Other genes	Maturase	*matK*					
	Envelope membrane protein	*cemA*					
	Subunit of acetyl-CoA	*accD*					
	C-type cytochrome synthesis gene	*ccsA*					
	Protease	*clpP*					
	Conserved hypothetical chloroplast reading frames	*ycf1*	*ycf2*	*ycf2-D2*			

### Comparative chloroplast genome analysis

**Figure 3:**
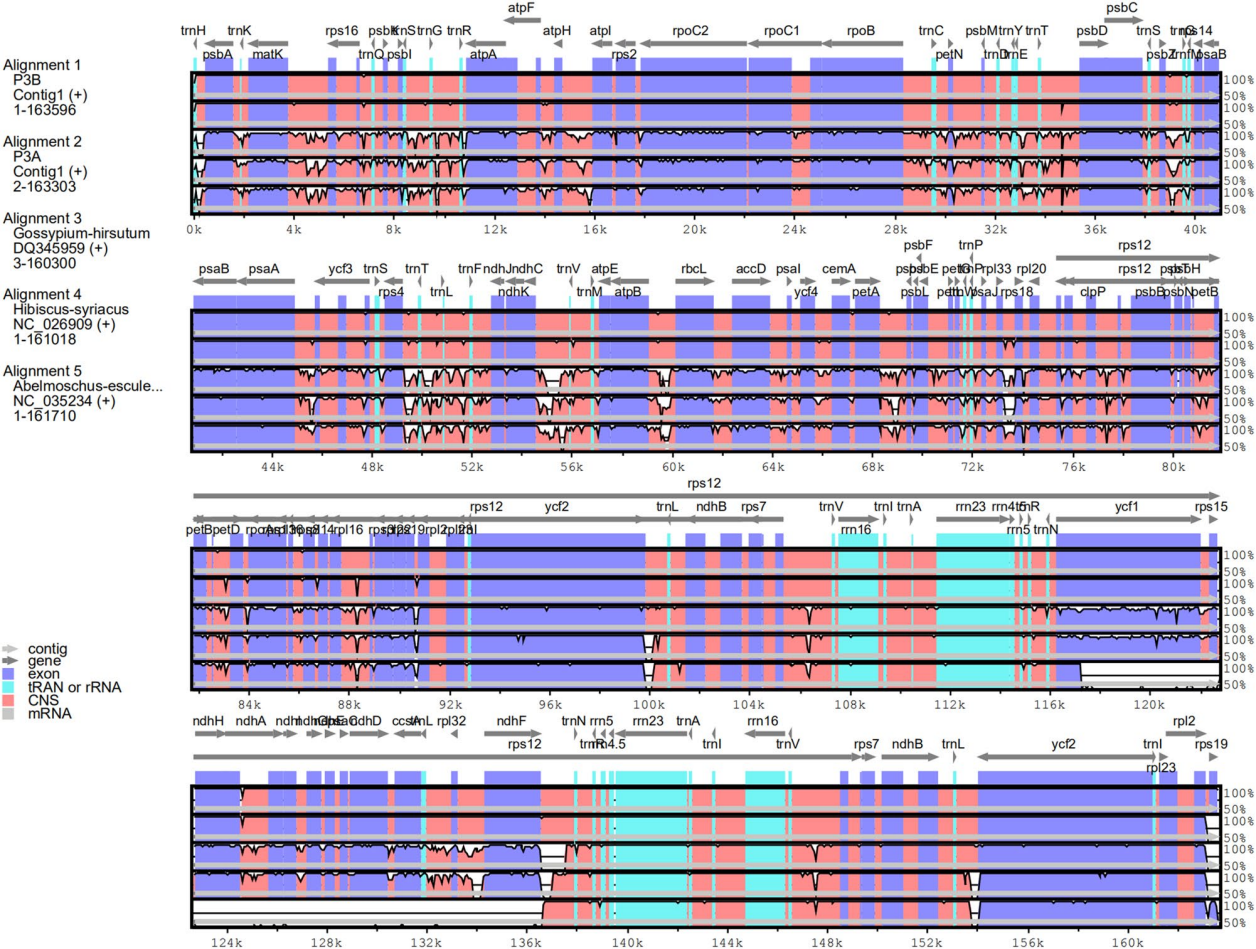
Comparison of the cp genome sequence of P3B, P3A, *Hibiscus syriacus Linn*, *Abelmoschus esculentus*, and *Gossypium hirsutum.*

**Figure 4:**
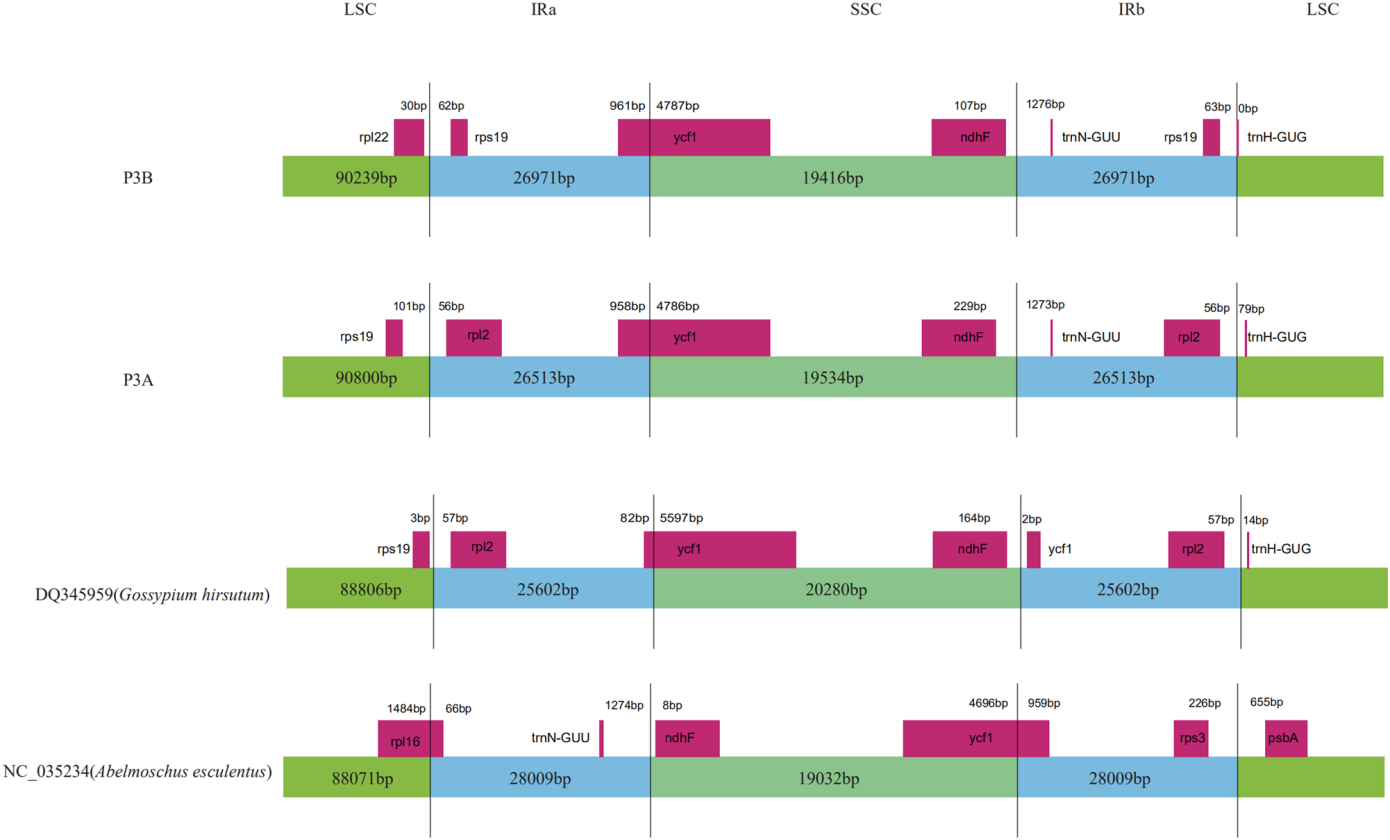
Comparison of the borders of the LSC, SSC, and IR regions of P3B, P3A, *Abelmoschus esculentus*, and *Gossypium hirsutum* cp genomes.

Although the coding region was found to be more highly conserved than the non-coding region, the coding region of kenaf chloroplast genome was still different from that of other three genomes (Fig. 3). The LSC-IRB-SSC-IRA boundary regions were compared within four closely related chloroplast genomes, P3B, P3A, *Abelmoschus esculentus*, and *Gossypium hirsutum* (Fig. 4). The *rps19* gene of P3B was located in IRa and IRb regions, while it was shifted to LSC region in P3A. The *ycf1* gene was extended from the IRa to the LSC region in P3B, P3A, and *Gossypium hirsutum*. However, *ycf1* was found in the junction of IRb/SSC in *Abelmoschus esculentus*. At the IRb/SSC boundary, the *ndhF* was observed with 107 bp, 229 bp, and 164 bp gap, respectively in P3B, P3A, and *Gossypium hirsutum*, which was located in SSC region of *Abelmoschus esculentus*. *trnN-GUU* gene was identified in IRb region with 1276 bp and 1273 bp apart from the IRb/LSC junctions of P3B and P3A, respectively, while was shifted to IRa region in *Abelmoschus esculentus*. *trnH-GUG* gene was at the junction of IRb/LSC in P3B and 79 bp apart from the IRb/LSC junction in P3A.

### Phylogenetic analysis

**Figure 5:**
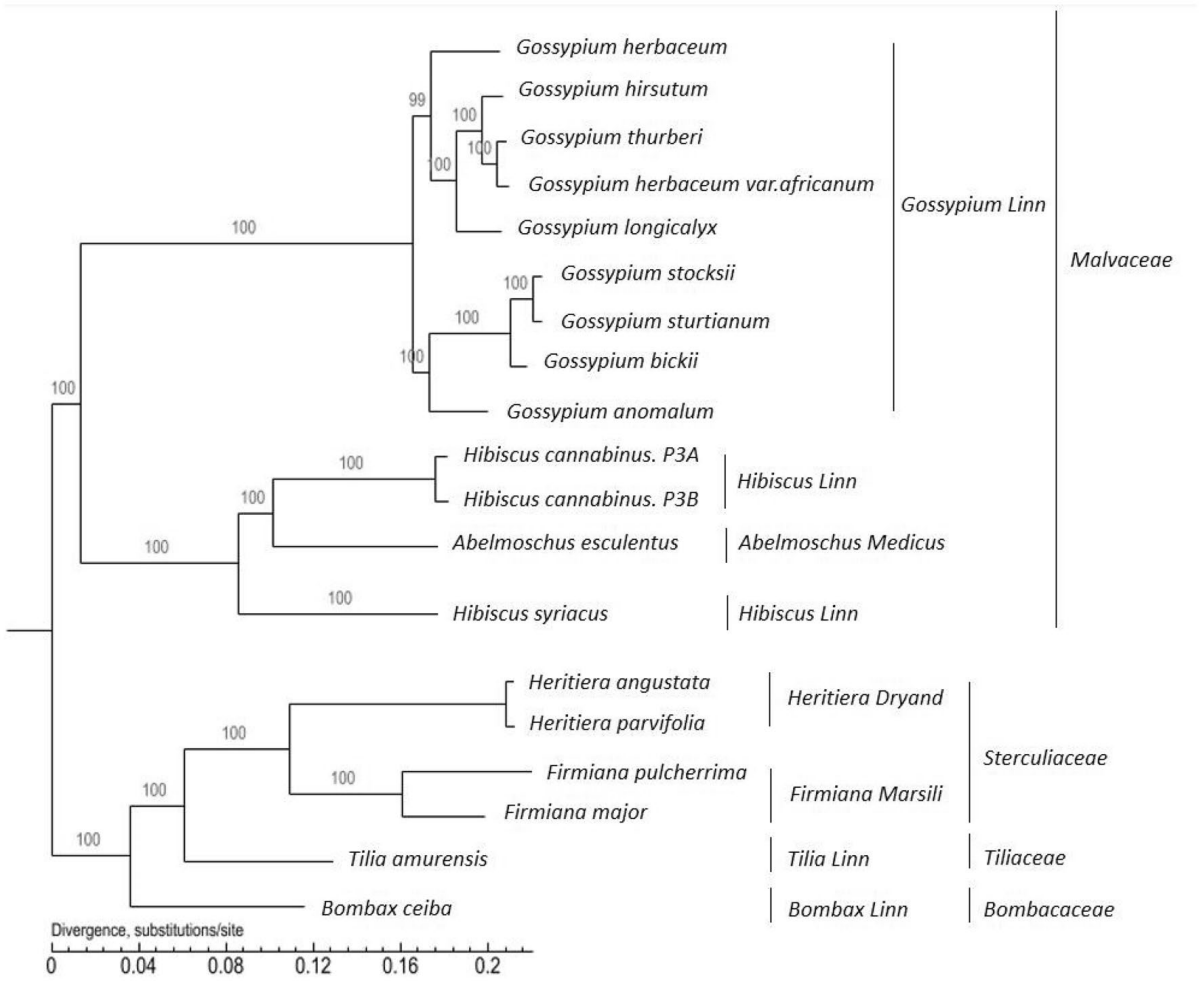
Molecular phylogenetic tree of 19 species of Malvales.

To analyze the phylogenetic position of kenaf within Malvales, 19 species of Malvales derived from four families, *Malvaceae*, *Sterculiacaea*, *Bombacacaea*, and *Tiliaceae* were aligned (Fig. 5). All 17 nodes were resolved well and reliable based on bootstrap value: 16 had bootstrap support of 100% and only 1 harbored the support of 99%. The phylogenetic tree showed that all the 19 species were classified into two clades. One clade included *Sterculiacaea*, *Bombacacaea*, and *Tiliaceae* families. Kenaf, *Abelmoschus esculentus*, and *Hibiscus syriacus* were clustered into the other clade, indicating that kenaf was more closely related to *Hibiscus syriacus* and *Abelmoschus esculentus* than cotton.

### SNPs analysis between P3A and P3B

**Figure 6:**
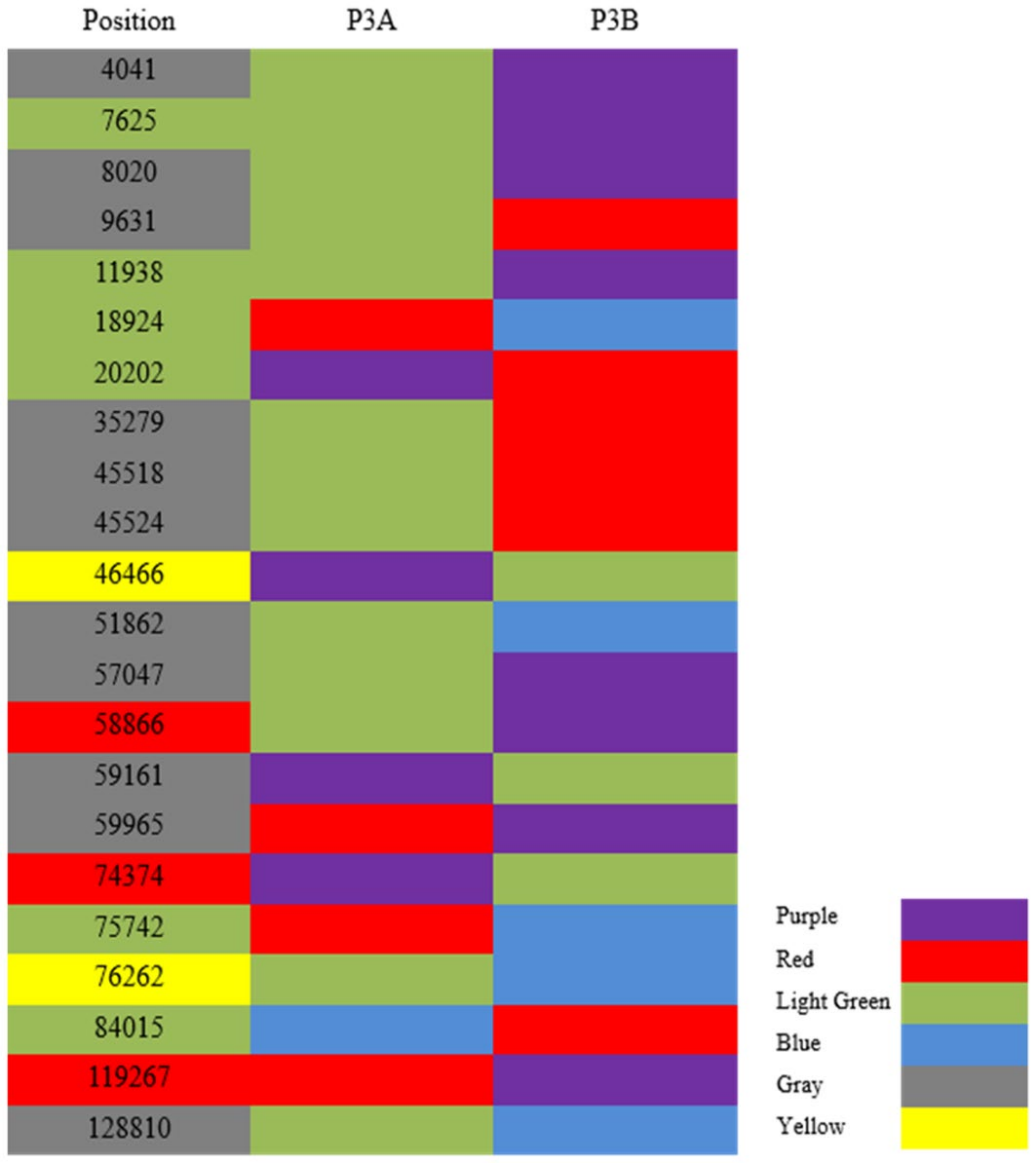
SNPs in gene coding region, gene intron and intergenic region between P3A and P3B. Note: DNA bases of P3A and P3B, guanosine (purple), thymine (red), adenine (light green), cytosine (blue). Position (red), nonsynonymous SNP in the CDS; Position (light green), synonymous SNP in the CDS; Position (yellow), SNP in the intron; Position (gray), SNP in the intergenic. Excel software was used for data processing and graph analysis.

**Figure 7:**
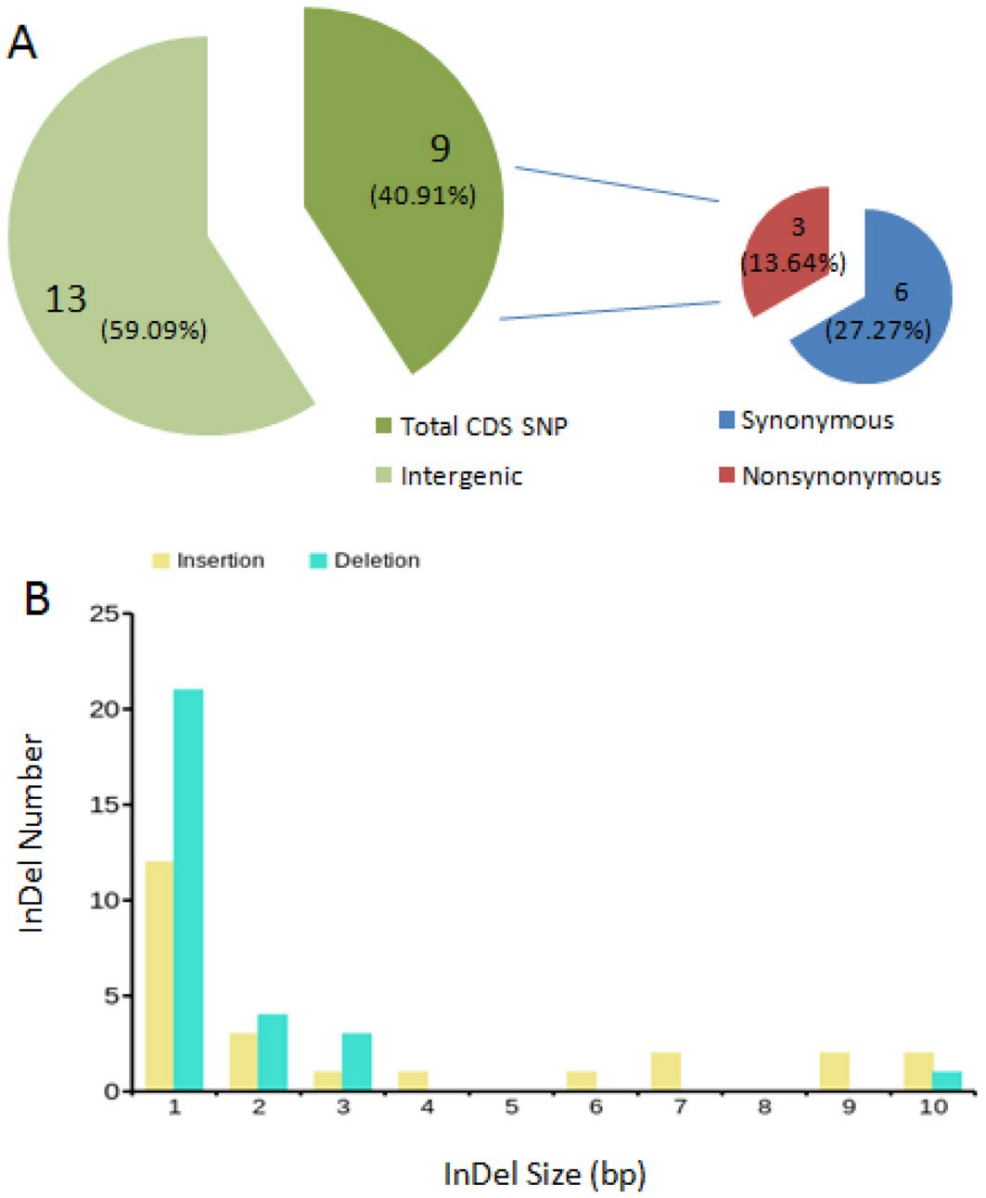
Statistical analysis of SNPs and Indels in P3A. (**A**) SNPs statistical analysis of P3A. (**B**) Indels statistical analysis. Excel and PPT software were used for data processing and graph analysis.

To detect the cpDNA differences between the CMS line P3A and its maintainer line P3B, setting P3B as a reference sequence, the two cp genomes were aligned for SNPs analysis. Although the chloroplast genomes of P3A and P3B showed high homology, structural variation still existed (Supplementary Fig. S2). Further analysis showed that a total of 22 SNPs were detected and located in gene coding region, gene intron region, and intergenic region (Fig. 6). As shown in Table 2, a total of 9 SNPs in gene coding region were located in *psbK*, *atpA*, *rpoC2*, *atpB*, *rpl20*, *clpP*, *rpoA*, and *ycf1*, including 6 synonymous SNPs and 3 nonsynonymous SNPs (Fig. 7A). Among these 3 nonsynonymous SNPs, the mutation of *atpB*, *rpl20*, and *ycf1* lead to amino acid changes. Moreover, 13 synonymous SNPs were found in intron and gene intergenic regions of P3A (Table 3). Of these, 2 SNPs were located in intron and 11 were in intergenic region.

**Table 2:** SNPs in gene coding region of P3A.

Position	Ref_base ↔ P3A_base	Codon mutate	aa mutate	Mutate type	Gene id	Position start	Position end
7,625	A ↔ G	TTA ↔ TTG	L ↔ L	Synonymous	*psbK*	7,548	7,733
11,938	A ↔ G	ACA ↔ ACG	T ↔ T	Synonymous	*atpA*	10,861	12,384
18,924	T ↔ C	TGT ↔ TGC	C ↔ C	Synonymous	*rpoC2*	17,859	22,040
20,202	G ↔ T	CTG ↔ CTT	L ↔ L	Synonymous	*rpoC2*	17,859	22,040
58,866	A ↔ G	AGT ↔ GGT	S ↔ G	Nonsynonymous	*atpB*	57,542	59,016
74,374	G ↔ A	AGG ↔ AAG	R ↔ K	Nonsynonymous	*rpl20*	74,280	74,633
75,742	T ↔ C	GCT ↔ GCC	A ↔ A	Synonymous	*clpP*	75,703	75,930
84,015	C ↔ T	GGC ↔ GGT	G ↔ G	Synonymous	*rpoA*	83,997	84,980
119,267	T ↔ G	TTT ↔ TTG	F ↔ L	Nonsynonymous	*ycf1*	116,250	121,997

**Table 3:** SNPs in gene intron and intergenic region of P3A.

Position	Reference base	P3A base	Gene start	Gene end	Gene location
46,466	G	A	45,753	47,879	Intron
76,262	A	C	75,703	77,909	Intron
4,041	A	G	2,208	3,722	Intergenic
8,020	A	G	8,182	8,292	Intergenic
9,631	A	T	10,861	12,384	Intergenic
35,279	A	T	35,399	36,460	Intergenic
45,518	A	T	45,753	47,879	Intergenic
45,524	A	T	45,753	47,879	Intergenic
51,862	A	C	52,785	53,261	Intergenic
57,047	A	G	57,117	57,518	Intergenic
59,161	G	A	57,542	59,016	Intergenic
59,965	T	G	60,171	61,625	Intergenic
128,810	A	C	128,533	128,778	Intergenic

### Indel analysis between P3A and P3B

**Figure 8:**
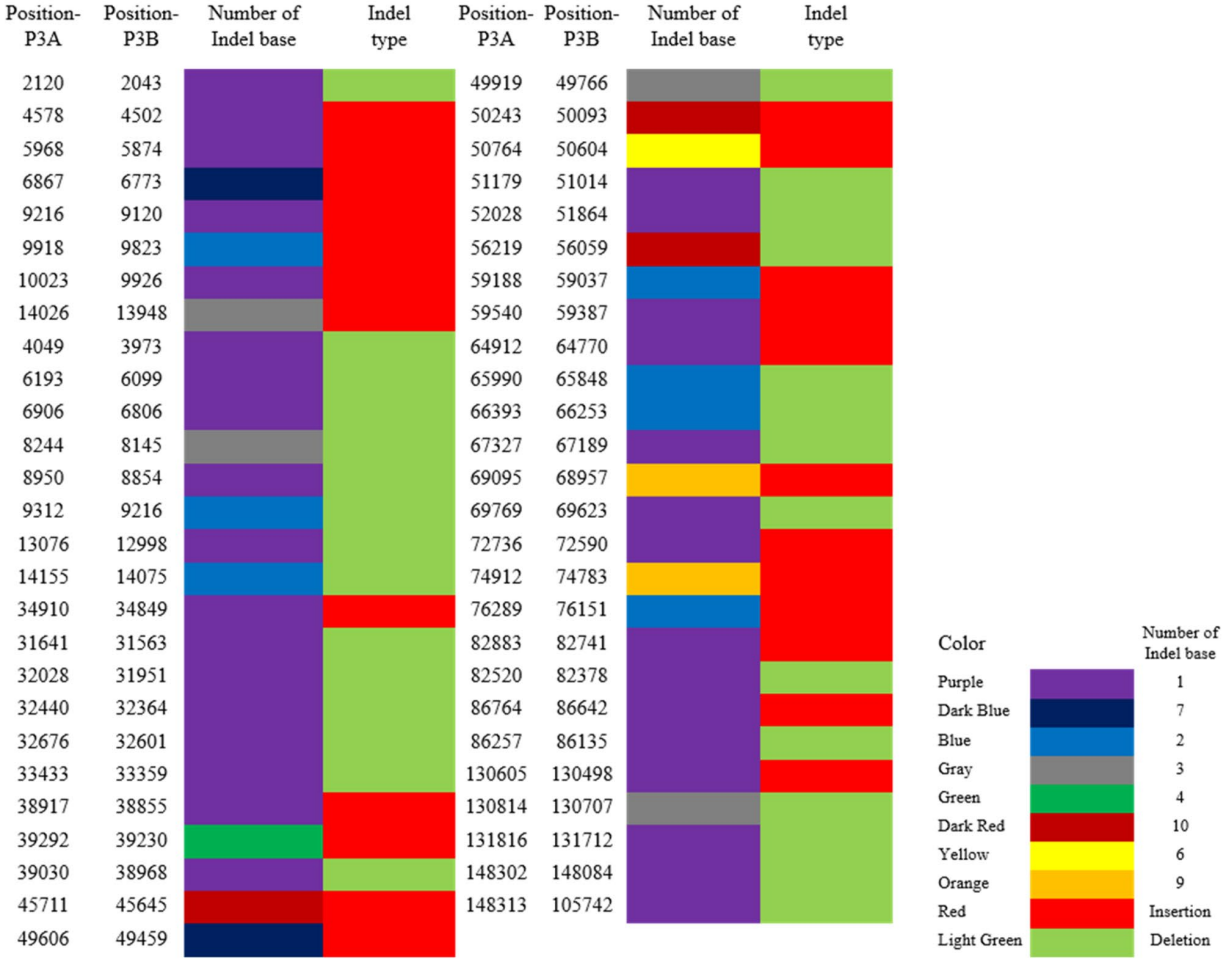
Indels in gene intron and intergenic regions between P3A and P3B. Excel software was used for data processing and graph analysis.

As for Indels, there were 53 Indel events, including 24 insertions and 29 deletions (Fig. 8) with different insertion and deletion size in P3A (Fig. 7B). All the 53 Indels were absent in the gene coding region and were distributed in the intergenic region of P3A (Table 4).

**Table 4:** Indel statistics between P3B and P3A.

Indel type	Sample Indel start	Sample Indel end	Ref start	Ref end	Indel sequence
Deletion	2,120	2,120	2,043	2,044	A
Insertion	4,578	4,579	4,502	4,502	A
Insertion	5,968	5,969	5,874	5,874	A
Insertion	6,867	6,874	6,773	6,773	TTAGAAT
Insertion	9,216	9,217	9,120	9,120	T
Insertion	9,918	9,920	9,823	9,823	TT
Insertion	10,023	10,024	9,926	9,926	T
Insertion	14,026	14,029	13,948	13,948	TTT
Deletion	4,049	4,049	3,973	3,974	A
Deletion	6,193	6,193	6,099	6,100	T
Deletion	6,906	6,906	6,806	6,807	A
Deletion	8,244	8,244	8,145	8,148	AAA
Deletion	8,950	8,950	8,854	8,855	A
Deletion	9,312	9,312	9,216	9,218	AA
Deletion	13,076	13,076	12,998	12,999	T
Deletion	14,155	14,155	14,075	14,077	TT
Insertion	34,910	34,911	34,849	34,849	T
Deletion	31,641	31,641	31,563	31,564	T
Deletion	32,028	32,028	31,951	31,952	A
Deletion	32,440	32,440	32,364	32,365	T
Deletion	32,676	32,676	32,601	32,602	A
Deletion	33,433	33,433	33,359	33,360	T
Insertion	38,917	38,918	38,855	38,855	A
Insertion	39,292	39,296	39,230	39,230	AAAT
Deletion	39,030	39,030	38,968	38,969	A
Insertion	45,711	45,721	45,645	45,645	AATAGAATTT
Insertion	49,606	49,613	49,459	49,459	ATAATAT
Deletion	49,919	49,919	49,766	49,769	AAA
Insertion	50,243	50,253	50,093	50,093	TATTTATTAT
Insertion	50,764	50,770	50,604	50,604	AAATAA
Deletion	51,179	51,179	51,014	51,015	A
Deletion	52,028	52,028	51,864	51,865	T
Deletion	56,219	56,219	56,059	56,069	TATATATATT
Insertion	59,188	59,190	59,037	59,037	TT
Insertion	59,540	59,541	59,387	59,387	T
Insertion	64,912	64,913	64,770	64,770	T
Deletion	65,990	65,990	65,848	65,850	TT
Deletion	66,393	66,393	66,253	66,255	AA
Deletion	67,327	67,327	67,189	67,190	T
Insertion	69,095	69,104	68,957	68,957	TAAATAGAG
Deletion	69,769	69,769	69,623	69,624	T
Insertion	72,736	72,737	72,590	72,590	T
Insertion	74,912	74,921	74,783	74,783	TTTTCTAGG
Insertion	76,289	76,291	76,151	76,151	TT
Insertion	82,883	82,884	82,741	82,741	T
Deletion	82,520	82,520	82,378	82,379	A
Insertion	86,764	86,765	86,642	86,642	T
Deletion	86,257	86,257	86,135	86,136	T
Insertion	130,605	130,606	130,498	130,498	A
Deletion	130,814	130,814	130,707	130,710	TTA
Deletion	131,816	131,816	131,712	131,713	A
Deletion	148,302	148,302	148,084	148,085	A
Deletion	148,313	148,313	105,742	105,743	T

### Verification of the high-throughput sequencing results

**Figure 9:**
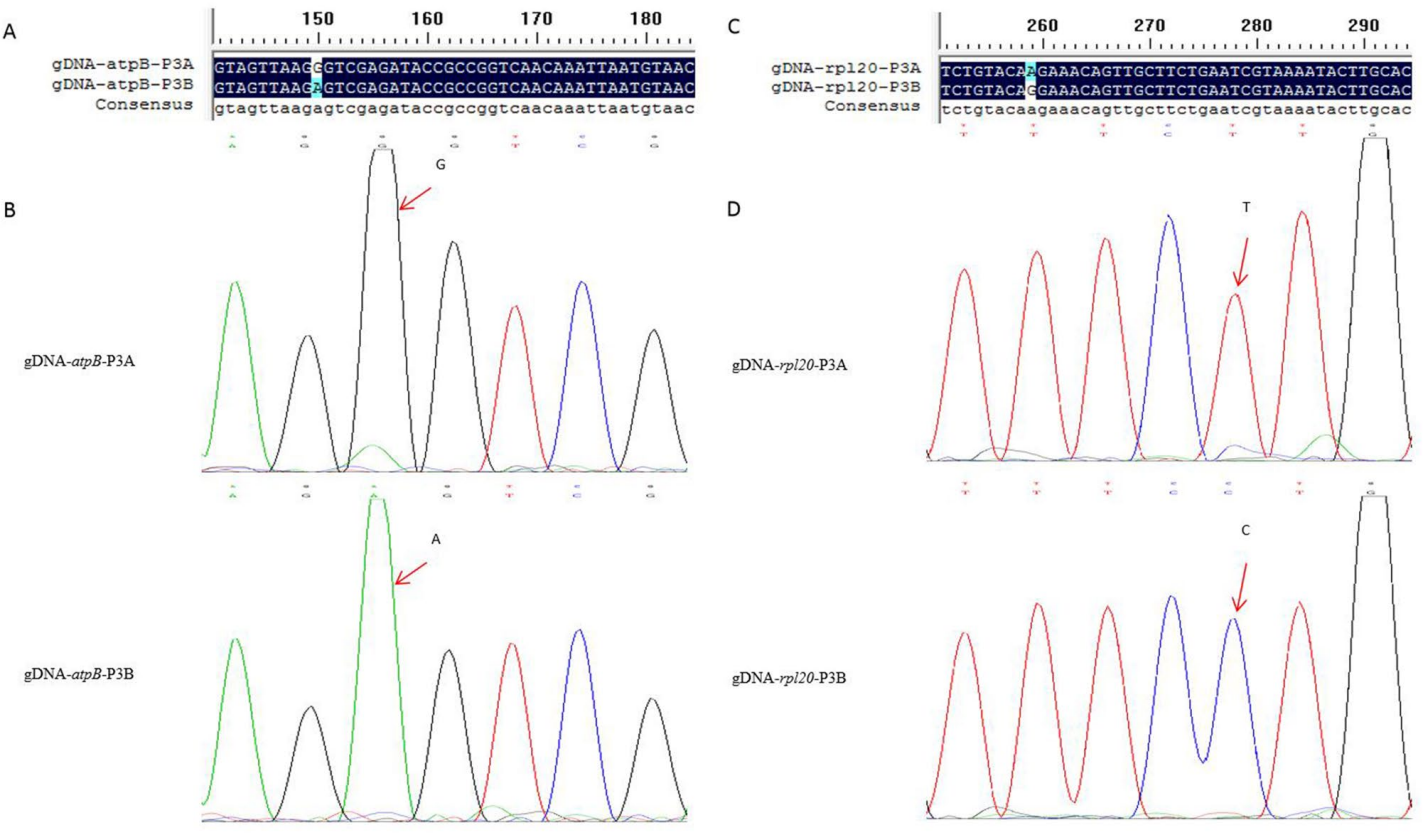
Cloning and sequencing results of *atpB* and *rpl20*. DNAMAN (V6.0.3.99 version) and PPT software were used for graph analysis.

To verify the accuracy of high-throughput sequencing results, two genes, *atpB* and *rpl20* were randomly selected for cloning and Sanger sequencing. Results showed that the sequences of *atpB* and *rpl20* existed differences between P3A and P3B at the DNA level. The differential bases were at the position of 151th bp of *atpB* and 260th bp of *rpl20* (Fig. 9A,C). Further analysis of the sequencing peak map exhibited that G-A and T-C mutation types were detected in *atpB* and *rpl20*, respectively (Fig. 9B,D). It was the same with the results listed in Table 2, inferring that the high-throughput sequencing results were reliable.

## Discussion

The chloroplast is a very important plant organelle that has its genome and plays a crucial role in generating energy through photosynthesis^9^. Chloroplast genome has been used as ideal research models, particularly for phylogeny^13^, DNA barcoding^14,15^, species conservation, and genome evolution^16^ because of the highly conservative structure. In the present study, we presented the complete nucleotide sequence of kenaf chloroplast genomes using the Illumina Hiseq and PacBio sequencing platforms. The chloroplast genomes of P3A and P3B were fully characterized. As shown in Figs. 1 and 2, the kenaf chloroplast genome was a typical circle DNA, similar to those from Malvaceae^13,17,18^. Moreover, the length of the chloroplast genome of kenaf P3B and P3A was 163,597 bp (Fig. 1) and 163,360 bp (Fig. 2), respectively. They were larger than those of Malvaceae plants^13,17,18^. In addition, a total of 131 and 132 genes, including 85 and 83 protein coding genes, 38 and 41 tRNA genes, and 8 rRNA and 8 rRNA were detected in P3B and P3A, respectively (Table 1). The gene number of kenaf was more than *Hibiscus syriacus* that also belonged to the *Hibiscus* genus and contained 81 protein-coding genes, 29 tRNA genes, and 4 rRNA genes^17^. The genome size differences within the species mentioned above might be due to the species differences.

Although the overall structure, genome size, gene number, and gene order of the chloroplast genome were conserved, the junctions between SSC and IR regions were usually different in the chloroplast genomes of higher plant. The border regions of LSC/IRa, IRa/SSC, SSC/IRb, and IRb/LSC were highly variable with many nucleotide variations in the chloroplast genomes of closely related species^19^. In this study, we compared the IR boundary regions of the chloroplast genome from three species. The organization of the kenaf chloroplast genome with a pair of IR regions separated by the SSC and LSC regions was the same with most sequenced angiosperm chloroplast genomes, emphasizing the highly conserved nature of plant chloroplast genomes^20^. However, the border of the kenaf chloroplast genome was a little different from that of other chloroplast genomes (Fig. 4), which probably contributed to the chloroplast genome size differences within Malvaceae species.

It is generally believed that mitochondrial genome rearrangement and generation of new open reading frames (ORFs) changed the transcription and translation products of mitochondrial DNA, resulting in CMS^21,22^. Unlike mitochondria, little attention has been paid to the relationship between plant chloroplast and CMS, especially in kenaf. Chloroplast, a special subcellular organelle, is closely linked to heterosis^23^ and may be involved in plant CMS^6,11,12^. Li and Liu reported that there were some relations between cpDNA and CMS in maize, rape, and wheat^24^. In the rice CMS line, Hou et al. found differential fragments between the CMS line and its maintainer using AFLP molecular marker technology^25^. Tang et al. revealed different SNPs and Indels models in the rice CMS line^6^. Recently, the chloroplast genome size and component between CMS-C cytoplasm and normal cytoplasm were highly consistent, but Indels or SNPs were also detected between the male sterile lines and maintainer lines of maize^26^. In our investigation, 22 SNPs and 53 Indels were found between the cp genomes of P3B and P3A, which were located in gene coding region, gene intron, and intergenic region (Tables 2, 3, 4). It was consistent with the previous studies mentioned above^6,26^. In particular, there were a total of 9 SNPs in the gene coding region, which were located in *psbK*, *atpA*, *rpoC2*, *rpl20*, *ycf1*, *atpB*, *clpP*, and *rpoA*, respectively. It was found that most of these genes were related to the photosynthetic system or photosynthesis. Furthermore, within the nonsynonymous SNPs, phenylalanine mutated to leucine in *ycf1*, serine changed to glycine in *atpB*, and arginine altered to lysine in *rpl20* (Table 2). Therefore, the cpDNA or chloroplast protein discrepancy might affect photosynthesis and energy metabolism and it was inferred that there might be some relationship between the chloroplast and kenaf CMS. CMS is the pollen abortion caused by nuclear–cytoplasm interaction^27^. Cytoplasmic genetic system included chloroplast and mitochondria. Nucleus, chloroplast, and mitochondria were not only independent, but also interrelated, infiltrated and influenced each other^28^. In the long-term evolution process, plants formed a coordinated relationship among the nucleus, chloroplast, and mitochondria, thus ensuring the normal growth and development of plants. However, once the coordination was broken during the pollen development, the normal information exchange between the nuclear and cytoplasm changed, then probably resulting in pollen abortion^29^. In other words, the coordination among the nucleus, chloroplast, and mitochondria of kenaf pollen cells might be broken due to the deviant cpDNA thus leading to the CMS of kenaf.

## Conclusions

We sequenced and characterized the chloroplast genomes of kenaf CMS line P3A and its maintainer line P3B. The bio-informatics comparison analysis of chloroplast genomes among Malvales was performed. SNP and Indel between the two lines were also detected, analyzed, and validated. Our findings revealed the differences in cpDNA between P3B and P3A, which provided basic information for the further study of kenaf CMS mechsnism.

## Materials and methods

### Sample collection

Kenaf CMS line P3A and its maintainer line P3B were used in the present study. Seeds of both the cultivars were sown and cultivated in half-strength Hoagland solution as described in our previous study^30^. Leaves from 25-day-old seedlings were collected and frozen with liquid nitrogen immediately.

### Chloroplast DNA (cpDNA) sequencing and genome assembly

Approximately 5 g of fresh leaves were harvested for DNA isolation using an improved extraction method^31^. After DNA isolation, 1 μg of purified DNA was fragmented and used to construct short-insert libraries according to the manufacturer’s instructions (Illumina), then sequenced on the Illumina Hiseq 4000 and PacBio platforms^32^.

Prior to assembly, raw reads were filtered. This filtering step was performed to remove the reads with adaptors, the reads showing a quality score below 20 (Q < 20), the reads containing a percentage of uncalled based (“N” characters) equal or greater than 10%, and the duplicated sequences. The chloroplast genome was reconstructed using a combination of de novo and reference-guided assemblies, and the following three steps were used to assemble chloroplast genomes^33^. First, the filtered reads were assembled into contigs using software SOAPdenovo2.04^34^. Second, contigs were aligned to the reference genome of *Hibiscus syriacus* (Accession: NC_026909.1) using BLAST, and aligned contigs (≥ 80% similarity and query coverage) were ordered according to the reference genome. Third, clean reads were mapped to the assembled draft chloroplast genome to correct the wrong bases, and the majority of gaps were filled through the local assembly.

### Genome annotation

The online DOGMA tool^35^ with default parameters was used to predict protein-coding genes, transfer RNA (tRNA) genes, and ribosome RNA (rRNA) genes. A whole chloroplast genome blast search (E-value ≤ 1e^−5^, minimal alignment length percentage ≥ 40%)^36^ was performed against 5 databases, including Kyoto Encyclopedia of Genes and Genomes (KEGG)^37–39^, Clusters of Orthologous Groups (COG)^40,41^, Non-Redundant Protein Database (NR), Swiss-Prot^42^, and Gene Ontology (GO)^43^ databases. The circular chloroplast genome maps of P3A and P3B were drawn using OrganellarGenomeDRAWv1.2^44^.

### Comparative chloroplast genome analysis

The complete chloroplast genomes of *Hibiscus cannabinus* (P3B and P3A) were compared with those of three other species, *Hibiscus syriacus Linn*, *Abelmoschus esculentus*, and *Gossypium hirsutum* using the mVISTA program in a Shuffle-LAGAN mode^45^. The contraction/expansion regions of the inverted repeat (IR) were compared among P3B, P3A, *Abelmoschus esculentus*, and *Gossypium hirsutum*. P3B was set as a reference for SNP and indel analysis between P3B and P3A.

### Phylogenetic analysis

The 19 completed chloroplast genome sequences representing Malvales plants were downloaded from the NCBI (Supplementary Table S5). Phylogenetic analysis was conducted based on maximum likelihood (ML) analysis using the general time-reversible invariant-sites (GTR + I) nucleotide substitution model with the default parameters in PhyML v3.0 (http://www.atgc-montpellier.fr/phyml/). The bootstrap probability of each branch was calculated by 1000 replications.

### SNP detection

The different sites between the sample sequence (P3A) and the reference sequence (P3B) were detected using MUMmer^46^ software. The potential SNP sites were checked through preliminary filtering. After that, the 100 bp sequences on both sides of the SNP sites of the reference sequence were extracted, and then BLAT^47^ (version: 35, http://genome.ucsc.edu) software was used and verified by comparing the extracted sequences with the assembly results. If the alignment length is less than 101 bp, it is considered as untrusted SNP, which will be removed. If the alignment is repeated many times, it is considered as repeated SNP, which will also be removed. Subsequently, BLAST^36^, RepeatMasker^48^, and TRF^49^ software were used to detect the repeated sequence area of the reference sequence, filter the SNP located in the repeated area, and finally obtain reliable SNP.

### Indel detection

Using LASTZ^50,51^ software, we compared the sample and reference sequences and the best alignment results were selected through the processing of axt_correction, axtsort and axtbest programs, and the preliminary Indel results were obtained. Then, 150 bp upstream and downstream of the Indel site of the reference sequence were compared with the sequencing reads of the sample by BWA^52^ (http://bio-bwa.sourceforge.net/) software and SAMtools^53,54^ (http://samtools.sourceforge.net/). Finally, the reliable Indel was obtained by filtration.

### DNA isolation and PCR validation

The leaves used for chloroplast sequencing were used for DNA extraction and PCR validation. DNA isolation was performed according to the CTAB protocol^55^ with minor modifications. PCR amplification was conducted according to the following procedures: initial denaturation at 95 °C for 3 min; 30 cycles of denaturation at 95 °C for 30 s, annealing at 50–60 °C for 1–2 min, extension at 72 °C for 1 min; and final extension at 72 °C for 5 min. The primer information was listed in Supplementary Table S6. For PCR reactions, each 25 μL reaction mixture contained 12.5 μL of 2 × Phanta Max Master Mix (Vazyme, China), 1.5 μL of primer (10 ppmol, forward primer and reverse primer each 0.75 μL), 1.5 μL of genomic DNA, and 9.5 μL of ddH_2_O. Then, PCR products were recycled, cloned, and sequenced.

## Supplementary Information

Supplementary Information.
