# Supplementary material for: Comparative analysis of chloroplast genomes of kenaf cytoplasmic male sterile line and its maintainer line
Source: Sci Rep. 2021 Mar 5;11:5301. doi: 10.1038/s41598-021-84567-1 (PMC7935921; doi:10.1038/s41598-021-84567-1)
Supplement: Supplementary file 1 — Supplementary Information. [file 41598_2021_84567_MOESM1_ESM.doc]

| Sample | Concentration（ng/μl） | OD  260/280 | OD  260/230 | Total DNA content（μg） |
| --- | --- | --- | --- | --- |
| P3A | 34.4 | 1.77 | 2.01 | 5.16 |
| P3B | 34.5 | 1.88 | 2.02 | 5.18 |
| **Table S1** DNA test results of kenaf. | | | | |

| Sample ID | Insert size (bp) | Raw data (Mb) | Clean data (Mb) | Reads length (bp) | Clean data Q20(%) | Clean data Q30(%) |
| --- | --- | --- | --- | --- | --- | --- |
| P3B | 430 | 6346 | 6071 | (150:150) | 98.44 | 95.31 |
| P3A | 299 | 3732 | 3471 | (-1:-1) | 97.73 | 94.12 |
| **Table S2** Sequencing data statistics. | | | | | | |

|  | | | | | | |
| --- | --- | --- | --- | --- | --- | --- |
| Sample | Genome size (bp) | Gene number (#) | Gene total length (bp) | Gene average length (bp) | Gene length/Genome (%) | GC Content (%) |
| P3B | 163,597 | 85 | 79,151 | 931 | 48.38 | 36.55 |
| P3A | 163,360 | 83 | 87,032 | 1,049 | 53.28 | 36.57 |
| **Table S3** Genome information statistics. | | | | | | |

|  | | | | | |
| --- | --- | --- | --- | --- | --- |
| Sample ID | ncRNA type | ncRNA number (#) | ncRNA total length (bp) | ncRNA average length (bp) | ncRNA length / Genome (%) |
| P3B | tRNA | 38 | 2,874 | 75 | 1.76 |
|  | rrn16 | 2 | 2,982 | 1,491 | 1.82 |
|  | rrn23 | 2 | 5,620 | 2,810 | 3.44 |
|  | rrn4.5 | 2 | 206 | 103 | 0.13 |
|  | rrn5 | 2 | 242 | 121 | 0.15 |
| P3A | tRNA | 41 | 3,051 | 74 | 1.87 |
|  | rrn16 | 2 | 2,982 | 1,491 | 1.83 |
|  | rrn23 | 2 | 5,620 | 2,810 | 3.44 |
|  | rrn4.5 | 2 | 206 | 103 | 0.13 |
|  | rrn5 | 2 | 242 | 121 | 0.15 |
| **Table S4** ncRNA statistics result. | | | | | |

| Species | Accession |
| --- | --- |
| *Abelmoschun esculentus* | NC-035234 |
| *Hibiscus syriacus Linn* | NC-026909 |
| *Gossypium thurberi* | GU907100 |
| *Gossypium hirsutum* | DQ345959 |
| *Gossypium stocksii* | NC-023218 |
| *Gossypium sturtianum* | JF317356 |
| *Gossypium longicalyx* | JF317354 |
| *Gossypium bickii* | JF317352 |
| *Gossypium herbaceum var.africanum* | HQ325742 |
| *Gossypium anomalum* | NC-023213 |
| *Gossypium herbaceum* | NC-023215 |
| *Bombax ceiba* | NC-037494 |
| *Firmiana pulcherrima* | NC-036395 |
| *Firmiana major* | NC-037242 |
| *Heritiera angustata* | NC-037784 |
| *Heritiera parvifolia* | NC-038057 |
| *Tilia amurensis* | MH169579 |
| P3B | MW446503 |
| P3A | MW446504 |
| **Table S5** 19 Malvales accessions used in this study. | |

| Primer | Sequences | Tm |
| --- | --- | --- |
| rpl20-F | AGAATTAAACGGGGATATATAGCTC | 55℃ |
| rpl20-R | TAATCTCGTTGGAAATCATATAAAG |
| atpB-S | TACTACTTCTGTTCCTGGAGTTTCC | 56℃ |
| atpB-A | TCCTTCACGAGTCCGTTCACC |
| **Table S6** Primers used in this study. | | |


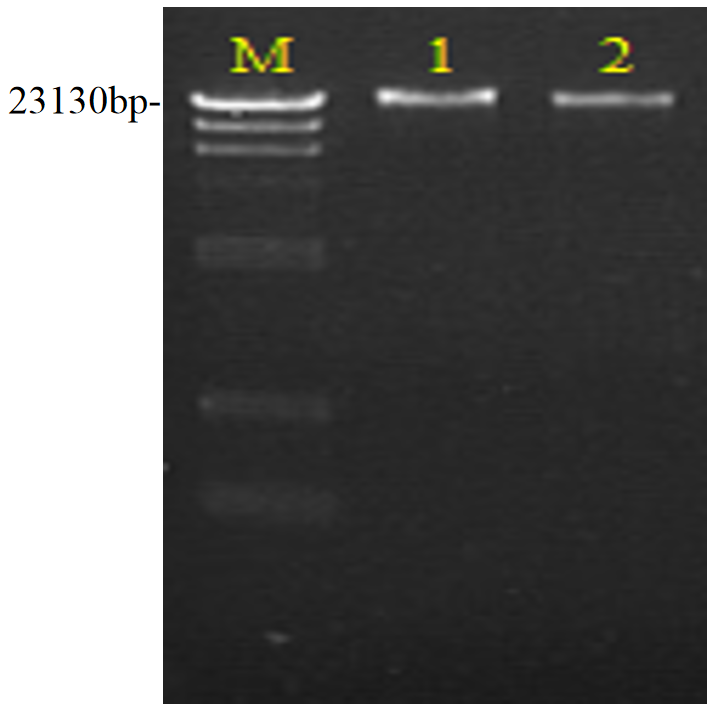


**Figure S1.** Results of DNA electrophoresis. Lane1, P3B; Lane2, P3A.


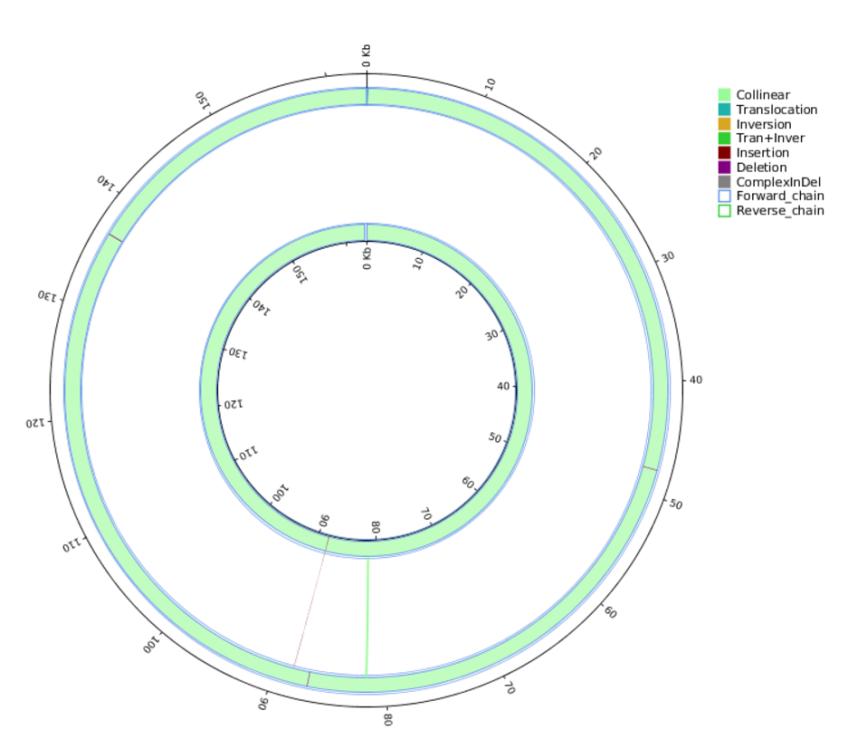


**Figure S2.** Map of structure variation of chloroplast genome. The inner circle is the reference genome and the outer circle is the sample genome.

**Title page**

**Comparative analysis of chloroplast genomes of kenaf cytoplasmic male sterile line and its maintainer line**

Danfeng Tang^1,2*†^, Fan Wei^1,2†^, Ruiyang Zhou^1*^

^1^College of Agriculture, Guangxi University, Nanning 530004, China

^2^Guangxi Key Laboratory of Medicinal Resources Protection and Genetic Improvement, Guangxi Botanical Garden of Medicinal Plants, Nanning 530023, China

†These authors contributed equally to this work.

* Correspondence: tdfmanuscript@163.com, ruiyangzhou@aliyun.com
